# *In silico* analysis and high-risk pathogenic phenotype predictions of non-synonymous single nucleotide polymorphisms in human *Crystallin beta A4* gene associated with congenital cataract

**DOI:** 10.1371/journal.pone.0227859

**Published:** 2020-01-14

**Authors:** Zhenyu Wang, Chen Huang, Huibin Lv, Mingzhou Zhang, Xuemin Li

**Affiliations:** 1 Department of Ophthalmology, Peking University Third Hospital, Beijing, China; 2 Beijing Key Laboratory of Restoration of Damaged Ocular Nerve, Peking University Third Hospital, Beijing, China; 3 Medical Research Center, Peking University Third Hospital, Beijing, China; University of Colorado Denver School of Medicine, UNITED STATES

## Abstract

In order to provide a cost-effective method to narrow down the number of pathogenic *Crystallin beta A4* (*CRYBA4*) non-synonymous single nucleotide polymorphisms (nsSNPs), we collected nsSNP information of the *CRYBA4* gene from SNP databases and literature, predicting the pathogenicity and possible changes of protein properties and structures using multiple bioinformatics tools. The nsSNP data of the *CRYBA4* gene were collected from 4 databases and published literature. According to 12 criteria, six bioinformatics tools were chosen to predict the pathogenicity. I-Mutant 2.0, Mupro and INPS online tools were used to analyze the effects of amino acid substitution on protein stability by calculating the value of ΔΔG. ConSurf, SOPMA, GETAREA and HOPE online tools were used to predict the evolutionary conservation of amino acids, solvent accessible surface areas, and the physical and chemical properties and changes of protein structure. All 157 *CRYBA4* nsSNPs were analyzed. Forty-four *CRYBA4* high-risk pathogenic nsSNPs (predicted to be pathogenic by all six software tools) were detected out of the 157 *CRYBA4* nsSNPs, four of which (c.283C>T, p.R95W; c.449T>A, p.V150D; c.475G>A, p.G159R; c.575G>C, p.R192P) should be focused on because of their high potential pathogenicity and possibility of changing protein properties. Thirty high-risk nsSNPs were predicted to cause a decrease of protein stability. Twenty-nine high-risk nsSNPs occurred in evolutionary conserved positions. Twenty-two high-risk nsSNPs occurred in the core of the protein. It is predicted that these high-risk pathogenic nsSNPs can cause changes in the physical and chemical properties of amino acids, resulting in structural changes of proteins and changes in the interactions between domains and other molecules, thus affecting the function of proteins. This study provides important reference value when narrowing down the number of pathogenic *CRYBA4* nsSNPs and studying the pathogenesis of congenital cataracts. By using this method, we can easily find 44 high-risk pathogenic nsSNPs out of 157 *CRYBA4* nsSNPs.

## Introduction

*Crystallin beta A4* (*CRYBA4*) is one of the pathogenic genes associated with congenital cataract. Mutations in these genes can cause abnormal lens metabolism in the embryonic stage and slow lens development and formation of opaque scar tissue in the lens, which eventually leads to congenital cataracts [1–3]. The annual incidence of congenital cataracts is 1.8–3.6/10,000 children [1], and inherited cataracts account for 8–25%. Among all hereditary modes, the autosomal dominant congenital cataract (ADCC) is the most common, accounting for about 73% [4–6] of cases. It has also been shown that variable clinical features are presented in different families with ADCC [7,8].

*CRYBA4* belongs to the crystallin family. Mammalian lens crystallins are divided into alpha, beta and gamma families. Alpha and beta families are further divided into acidic and basic groups. *CRYBA4*, a beta acidic group member, is part of a gene cluster along with beta-B1 (CRYBB1), beta-B2 (CRYBB2) and beta-B3 (CRYBB3). The human *CRYBA4* gene is located in chromosome 22 (22q12.1) and contains seven exons. There is one *CRYBA4* transcript (NM_001886.3) and one protein product (NP_001877.1). During the development of the human eye, CRYBA4 is one of several crystallins expressed in mature lens fiber cells, constitutes 5% of the proteins of vertebrate eye lens, and maintains the transparency and refractive index of the lens [9,10]. CRYBA4 is higher in neonatal lenses than adult lenses [11]. Therefore, mutations of *CRYBA4* may lead to visual impairment of neonates.

In the human genome, single nucleotide polymorphisms (SNPs) account for more than 90% of all nucleic acid sequence variations. Non-synonymous single nucleotide polymorphisms (nsSNPs) in the protein coding region cause amino acid substitution, which may affect protein function and lead to pathogenic phenotypes [12]. In 2006, the first mutation in the *CRYBA4* gene (c.242T>C, p.L69P) was found and was reported to be associated with the autosomal dominant congenital cataract [11]. Our team also found in 2018 that two heterozygous mutations of the *CRYBA4* gene might be pathogenic mutations in the Chinese autosomal dominant congenital cataract families. So far, six nsSNPs of the *CRYBA4* gene associated with congenital cataracts have been confirmed (A9V, G64W, Y67N, L69P, S93P, F94S) [11,13–17].

It has been speculated there are more ADCC-related *CRYBA4* nsSNPs that have not been found. It is necessary to seriously study the relationship between *CRYBA4* nsSNPs and the *CRYBA4* gene pathogenic phenotype, which will help analyze the pathogenesis of ADCC. With current expanding applications of next-generation sequencing and increasingly developed computational methods, the preliminary pathogenicity screening of gene mutation sites based on *in silico* analysis using bioinformatics tools has been widely recognized and applied by researchers. The performances of different bioinformatics tools under various conditions are different. Li. et al [18] compared 12 performance measures of 23 methods based on three independent benchmark datasets. The 12 criteria included positive predictive value (PPV), negative predictive value (NPV), false negative rate (FNR), sensitivity, false positive rate (FPR), specificity, Mathew correlation coefficient (MCC), receiver operating characteristic (ROC) curve, area under the curve (AUC), high-sensitivity regional AUC (hser-AUC) and high-specificity regional AUC (hspr-AUC). In this study, we systematically collect the nsSNP information of the *CRYBA4* gene from SNP databases and literature, screening the nsSNPs for high-risk pathogenicity using multiple bioinformatics software tools based on their high accuracy and frequency of use [18,19]. This study is undertaken to explore the nsSNPs of the *CRYBA4* gene responsible for ADCC and to predict the deleterious nature of the mutation.

## Materials & methods

### Nonsynonymous SNP data retrieval

The nsSNP data were retrieved from four databases, including the Single Nucleotide Polymorphism database (dbSNP, RRID: SCR_002338, http://www.ncbi.nlm.nih.gov/projects/SNP/) [20], Clinvar database (RRID: SCR_006169, https://www.ncbi.nlm.nih.gov/clinvar) [21], the Human Gene Mutation Database (HGMD, RRID: SCR_001888, http://www.hgmd.cf.ac.uk/ac/index.php) [22] and DisGeNET database (RRID: SCR_006178, http://www.disgenet.org) [23]. In order to avoid omitting additional nsSNPs from the literature, we used “CRYBA4”, “nsSNP” and “congenital cataract” as search terms to retrieve data. The relevant information of the *CRYBA4* nsSNP included SNP ID, chromosome loci, nucleic acid changes, amino acid changes and whether they were reported. The information on mRNA (NM_002473.5) and protein (NP_002464.1) was obtained from the National Center for Biotechnology Information (NCBI, http://www.ncbi.nlm.nih.gov). All authors didn’t have access to information that could identify individual participants during or after data collection. Choosing pathogenicity predictors in our study was decided based on high accuracy and frequency of use [18].

### Prediction of pathogenicity of nsSNPs

To analyze and predict the pathogenicity of *CRYBA4* nsSNPs precisely, six software tools were used including Mutpred2 (RRID: SCR_010778, http://mutpred.mutdb.org/) [24], PANTHER-PSEP (PANTHER Evolutionary analysis of coding SNPs, RRID: SCR_005145, http://pantherdb.org/tools/csnpScoreForm.jsp) [25], PhD-SNP (RRID: SCR_010782, http://snps.biofold.org/phd-snp/phd-snp.html) [26], PolyPhen 2.0 (Polymorphism phenotyping 2.0, RRID: SCR_013189, http://genetics.bwh.harvard.edu/pph2/) [27], PROVEAN (Protein variation effect analyzer, RRID: SCR_002182, http://provean.jcvi.org/index.php) [28], and SIFT (Sorting intolerant from tolerant, RRID: SCR_012813, http://sift.bii.a-star.edu.sg/) [29].

Mutpred2 is a sequence-homology based bioinformatics tools predicting potential pathogenicity and changes of protein properties. An amino acid substitution with the general score of >0.5–1 was considered to be pathogenic.

PANTHER-PSEP is able to calculate the score of position-specific evolutionary preservations. It measures the length of time (in millions of years) a position in a current protein has been preserved by tracing back to its reconstructed direct ancestors. The longer a position has been preserved, the more likely that it will have a deleterious effect. The thresholds were: "probably damaging" (time > 450my, corresponding to a false positive rate of ~0.2 as tested on HumVar), "possibly damaging" (450my > time > 200my, corresponding to a false positive rate of ~0.4) and "probably benign" (time < 200my).

PhD-SNP is a pathogenicity-predicting tool based on support vector machines. The Swiss-prot database was used for training and the prediction was used with 20-fold cross validation.

PolyPhen 2.0 is a widely used supervised machine-learning Na ve Bayes classifier. The two algorithms within PolyPhen2 were HumDiv and HumVar. The cut off score was 0.50 and mutations with scores over 0.50 were predicted to be pathogenic. HumDiv was trained using all 3,155 damaging alleles annotated in the UniProt database as causing human Mendelian diseases and affecting protein stability or function, together with 6,321 differences between human proteins and their closely related mammalian homologs, assumed to be non-damaging. HumVar was trained using all 13,032 human disease-causing mutations from UniProt and 8,946 human nsSNPs without annotated involvement in disease, which the operators treated as non-damaging. The operators of PolyPhen2 indicated that HumVar had lower accuracy than HumDiv. One reason was that nsSNPs assumed to be non-damaging in the HumVar dataset included a sizable fraction of mildly deleterious alleles that may not be fixed in the evolving lineage. Also, the HumDiv dataset used extra criteria to avoid possible erroneous annotations of damaging mutations. Therefore, we chose the predicting results of HumDiv [27].

PROVEAN and SIFT are sequence homology-based tools which have independently developed algorithms. The cutoff score of PROVEAN is -2.5 and mutations with scores over -2.5 are predicted to be pathogenic. The cutoff score of SIFT is 0.05 and mutations with scores over 0.05 are predicted to be pathogenic.

In order to summarize the predictive results of the software tools, we set a scoring criterion. If a nsSNP was predicted to be “benign” or “harmless” by one of the six software tools, it would score 0. However, it would get 1 point if one software tool predicted it as a “pathogenic” or “harmful” nsSNP. The nsSNP that was predicted to be “pathogenic” by all six software tools would be defined as a “*CRYBA4* high-risk pathogenic nsSNP” with 6 points.

### Prediction of protein stability

Protein stability is the basic characteristic that affects the function, activity and regulation of biological molecules. Free energy of protein unfolding is a key index of protein stability. By analyzing the influence of mutation on free energy, the effect of mutation on protein stability could be accurately determined. I-Mutant 2.0 (http://folding.biofold.org/i-mutant/i-mutant 2.0) [30], Mupro (http://mupro.proteomics.ics.uci.edu/) [31] and INPS (Impact of Non-synonymous mutations on Protein Stability, http://inpsmd.biocomp.unibo.it) [32]. In the case of unknown protein structure, the online software tools could calculate and predict whether the substitution could change the stability of protein by using support vector machines (SVM) with the amino acid sequence and amino acid substitution infomration. The software predicted the change of stability by calculating the change of thermodynamic free energy (ΔΔG) and the direction of change after single point mutation of protein: ΔΔG > 0 indicated stabilization while a negative value indicated destabilization.

### Evolutionary conservation analysis of nsSNPs and prediction of protein structure and property changes

The *CRYBA4* domain was retrieved using the Uniprot database (https://www.uniprot.org/). ConSurf online software (http://consurf.tau.ac.il) [33] was used to analyze the evolutionary conservation of amino acids by calculating the conservation score through unique algorithm. The amino acids with scores between 7 and 9 were evolutionary conservative amino acids. The SOPMA online software (https://npsa-prabi.ibcp.fr/cgi-bin/npsa_automat.pl?page=npsa_sopma.html) [34] used five independent algorithms to predict the secondary structure of proteins. Then, the GETAREA online software (http://curie.utmb.edu/getarea.html) [35] was used to predict the effect of amino acid mutation by calculating the solvent accessible surface areas of the proteins. The HOPE online software (Have (y) Our Protein Explained, http://www.cmbi.ru.nl/hope/input/) [36] was used to predict the effect of amino acid mutation on physical and chemical properties, hydrophobicity, spatial structure and function of proteins.

## Results

### Collection and collation of nsSNP data

**Fig 1:**
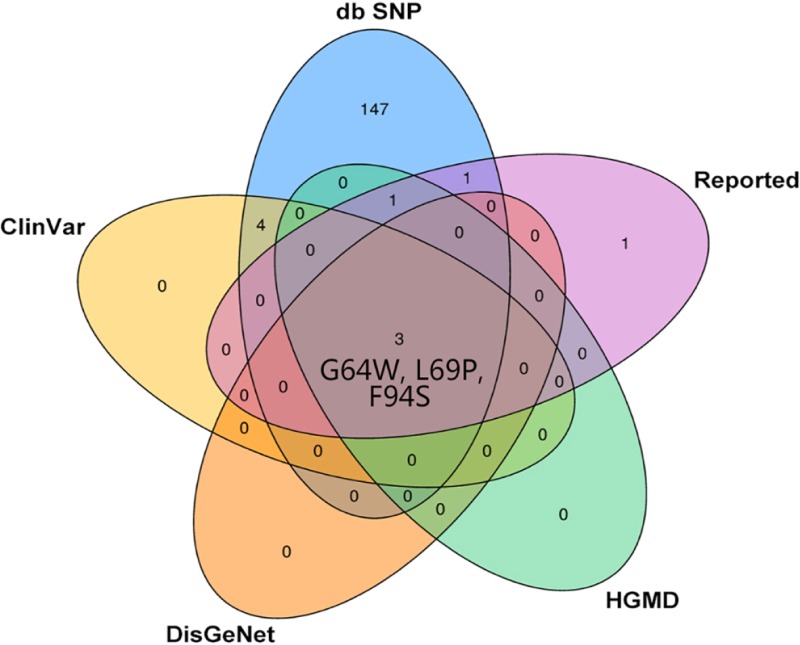
CRYBA4 coding region nsSNP dataset intersection Wayne map. The information of the Wayne map is obtained from db SNP database, ClinVar database, HGMD database, DisGeNET database and literature report preliminary screening.

We retrieved 176 nsSNPs related to the *CRYBA4* gene from four databases (155 from the db SNP database, seven from the ClinVar database, five from the HGMD database, and three from the DisGeNET database). After manual screening and eliminating duplicate records, there were 156 *CRYBA4* nsSNPs included in four databases. It is noteworthy that five (A9V, G64W, Y67N, L69P, F94S) of the 156 nsSNPs in various databases have been reported to be associated with congenital cataracts. There is one reported nsSNP (c. 277T>C, p. S93P) that has not yet been included in any database (Fig 1). According to the results of database screening and literature reports, there are only three nsSNPs (G64W, L69P, F94S) that have been reported in four databases and in the literature.

### Prediction of pathogenicity of nsSNPs

**Fig 2:**
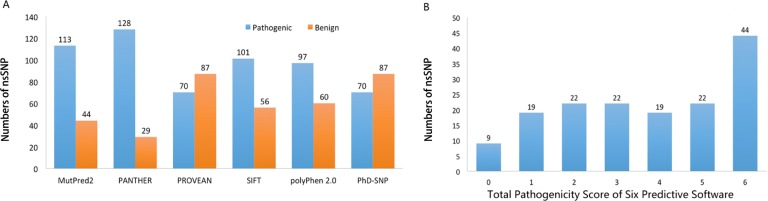
Prediction of pathogenicity of nsSNPs by Mutpred2, PANTHER-PSEP, PhD-SNP, PolyPhen 2.0, PROVEAN and SIFT software. A. The amount of "pathogenic" or "benign" nsSNPs predicted by each bioinformatics tool. B. Number of nsSNPs with different pathogenicity scores of six bioinformatics tools.

Six pathogenicity prediction software tools (Mutpred2, PANTHER-PSEP, PhD-SNP, PolyPhen 2.0, PROVEAN and SIFT) were used to predict the pathogenicity of 157 nsSNPs. Fig 2A shows the prediction results of six computation tools. As a result of integrating six software tools, nine nsSNPs were predicted to be "benign" with a score of 0, while 19, 22, 22, 19 and 22 nsSNPs got a score of 1, 2, 3, 4 and 5, respectively, for "pathogenic" or "harmful" (Fig 2B). There were 44 nsSNPs that were predicted to be "pathogenic" or "harmful" by all the six software tools and received 6 points. We defined these nsSNPs as "*CRYBA4* high-risk pathogenic nsSNPs". As shown in Table 1, these 44 nsSNPs can lead to the substitution of 39 amino acids in the CRYBA4 amino acid sequence. Among these nsSNPs, four nsSNPs (G64W, L69P, S93P, F94S) have been reported to be associated with congenital cataracts. The other 40 nsSNPs are the newly identified high-risk pathogenic nsSNPs.

**Table 1:** CRYBA4 high-risk pathogenic nsSNPs that were predicted to be "pathogenic" or "harmful" by all the six pieces of software.

snpid	Nucleic acid change	Amino acid change	MutPred2 score	PANTHER-PSEP preservation time	PhD-SNP prediction	PolyPhen 2.0 score	PROVEAN score	SIFT score
rs1173547883	28G>A	G10R	0.846	750	Disease	0.999	-2.77	0.003
rs760976886	34T>A	W12R	0.852	457	Disease	0.86	-3.7	0
rs1435054387	64T>C	F22L	0.887	750	Disease	0.717	-5.2	0.013
rs776802540	73C>T	R25W	0.67	750	Disease	1	-5.88	0
rs150427830	76C>T	R26W	0.69	750	Disease	0.58	-4.83	0.03
rs12053788	77G>C	R26P	0.879	750	Disease	0.994	-4.27	0.014
rs762531871	82G>A	E28K	0.905	750	Disease	0.977	-3.6	0.007
rs765421650	94G>A	E32K	0.676	324	Disease	0.786	-3.04	0.015
rs758467634	98G>A	C33Y	0.894	750	Disease	1	-9.98	0.043
rs773249225	134G>T	R45L	0.895	457	Disease	0.751	-4.87	0.045
rs1057524710	137C>A	S46Y	0.939	750	Disease	1	-5.63	0
rs1332414078	167G>A	G56D	0.842	750	Disease	1	-5.21	0.001
rs1447609778	172G>A	E58K	0.911	750	Disease	1	-3.92	0.001
rs1114167427	190G>T	G64W	0.937	1628	Disease	1	-7.94	0
rs74315487	206T>C	L69P	0.956	750	Disease	1	-6.52	0
rs778397499	212G>C	R71L	0.654	457	Disease	0.805	-4.58	0.001
	212G>T	R71P	0.83	457	Disease	0.953	-3.79	0.001
rs200572268	217G>A	E73K	0.816	750	Disease	0.685	-3.5	0.003
rs760857239	257A>G	Y86S	0.508	750	Disease	0.999	-5.11	0.034
	277T>C	S93P	0.921	750	Disease	1	-4.59	0.001
rs74315486	281T>C	F94S	0.803	457	Disease	1	-5.17	0.001
rs1459497417	283C>T	R95W	0.766	750	Disease	1	-7.71	0
rs749066010	284G>T	R95L	0.833	750	Disease	1	-6.75	0
rs751201974	311A>G	D104G	0.555	324	Disease	0.712	-4.49	0.005
rs140200694	331G>A	E111K	0.919	750	Disease	1	-3.83	0.001
rs760225068	413A>G	E138G	0.71	750	Disease	0.948	-3.71	0.024
rs866288374	422C>T	S141F	0.931	750	Disease	1	-5.72	0
rs1398882909	440G>T	G147V	0.945	1628	Disease	1	-8.71	0
rs765296550	443C>A	A148D	0.783	457	Disease	1	-3.08	0.002
rs1194205126	449T>A	V150D	0.957	750	Disease	1	-6.84	0
rs1180663561	452G>T	C151F	0.809	457	Disease	1	-2.91	0.037
rs780358100	464C>T	P155L	0.868	750	Disease	1	-9.71	0.009
rs755086807	467G>A	G156D	0.911	750	Disease	1	-6.85	0.002
rs1000021247	475G>A	G159R	0.951	751	Disease	1	-7.81	0
rs1168471465	483G>T	Q161H	0.826	750	Disease	1	-4.86	0
rs1299110590	485A>G	Y162C	0.931	750	Disease	1	-8.17	0
rs764908395	511G>A	G171S	0.876	750	Disease	1	-5.39	0.002
rs1162676984	518A>G	Y173C	0.932	750	Disease	1	-7.23	0
rs201666412	529C>T	R177W	0.54	361	Disease	0.998	-4.27	0.007
rs1237740955	532G>A	E178K	0.86	455	Disease	0.946	-3.5	0.002
rs1472168422	535T>C	W179R	0.906	1628	Disease	0.973	-9.35	0
rs758790937	575G>A	R192H	0.607	750	Disease	1	-4.66	0
	575G>C	R192P	0.878	750	Disease	1	-6.51	0
rs752825164	c.574C>T	R192C	0.743	750	Disease	1	-7.45	0

The bold black font represents the nsSNP reported in the literature. The cutoff score of Mutpred2 is 0.50 and mutations with scores over 0.50 are predicted to be pathogenic. The thresholds of PANTHER-PSEP were: "probably damaging" (time > 450my, corresponding to a false positive rate of ~0.2 as tested on HumVar), "possibly damaging" (450my > time > 200my, corresponding to a false positive rate of ~0.4) and "probably benign" (time < 200my). The cut off score of PolyPhen2 is 0.50 and mutations with scores over 0.50 are predicted to be pathogenic. The cutoff score of PROVEAN is -2.5 and mutations with scores over -2.5 are predicted to be pathogenic. The cutoff score of SIFT is 0.05 and mutations with scores over 0.05 are predicted to be pathogenic.

The other two reported pathogenic nsSNPs were Y67N and A9V. Y67N got 5 points and only PANTHER predicted it as a “benign” nsSNP. A9V was predicted to be "benign" by all six software tools and scored 0 points. The predictions above indicate that the accuracy when integrating the six pieces of predicting software reaches 66.67% (4/6), although there is a certain false negative rate in the results due to insufficiencies of the predicting algorithm of each software.

### Prediction of protein stability

In order to predict the effects of amino acid substitution caused by nsSNP on protein stability, we used I-Mutant 2.0, Mupro, INPS online software and the CRYBA4 protein (NP_001877.1) information to compare the effects of mutant and wild type amino acids on protein free energy. Among 157 nsSNPs, I-Mutant 2.0 predicted that 126 amino acid substitutions caused by nsSNPs resulted in the decrease of protein free energy. Mupro software and INPS software predicted 148 and 123 mutations, respectively, leading to a decrease in protein stability. When integrating the predicted results, all three software tools predicted that the free energy of 105 sites of protein would decrease after the mutation of amino acids and the ΔΔG < 0, resulting in protein stability decline. Among these, there were six sites (V15A, R26P, F94S, Y126S, V163A and W179R) that were scored below –1 by all three software tools, indicating that the stability of protein decreased sharply after the mutations.

**Fig 3:**
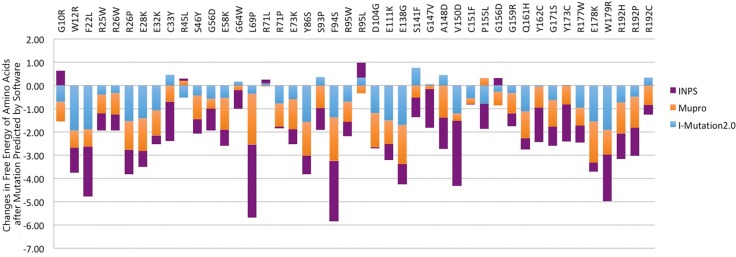
I-Mutant 2.0, Mupro and INPS software predicted the change of protein free energy caused by nsSNPs.

As shown in Fig 3, 44 "*CRYBA4* high-risk pathogenic nsSNPs" can cause 39 wild-type amino acids to be replaced by 44 mutant amino acids. Three software tools (I-Mutant 2.0, Mupro and INPS) suggest that 30 of the 44 nsSNPs may lead to a decline in protein stability. Among the 30 nsSNPs, there are 28 newly discovered high-risk pathogenic nsSNPs: W12R, F22L, R25W, R26W, R26P, E28K, E32K, S46Y, G56D, E58K, L69P, R71P, E73K, Y86S, F94S, R95W, D104G, E111K, E138G, V150D, C151F, G159R, Q161H, Y162C, G171S, R177W, E178K, W179R, R192H, and R192P. The two nsSNPs, L69P and F94S, were previously reported pathogenic variants identified in families.

### Evolutionary Conservation Analysis and Protein Structure Analysis

**Fig 4:**
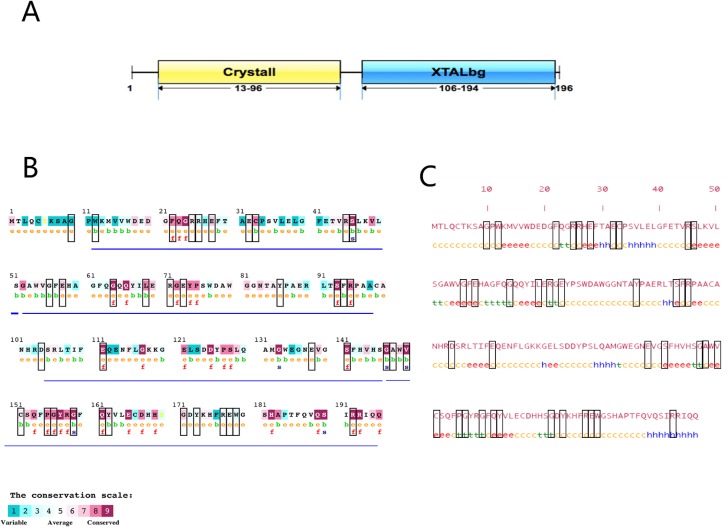
Structural domains, evolutionary conservation and protein structure analysis of CRYBA4 high-risk pathogenic nsSNPs. A. CRYBA4 protein domain. CRYBA4 consists of "Crystall" and "XTALbg" domains. There are 84 amino acids in Crystall domain (from 13th to 96th) and 89 amino acids in XTALbg domain (from 106th to 194th). B. ConSurf software CRYBA4 protein amino acid evolutionary conservation prediction results. The black boxes indicate wild amino acids that would be affected by CRYBA4 high-risk pathogenic nsSNPs. Four motifs are marked as blue underlines. Evolutionary conserved sites get scores between 7 and 9. The letter “e” denotes the exposed residues according to the neural-network algorithm; the letter “b” denotes the buried residues according to the neural-network algorithm; the letter “f” denotes the predicted functional residues (highly conserved and exposed) and the letter “s” denotes the predicted functional residues (highly conserved and buried). C. Prediction of secondary structure of CRYBA4 protein by SOPMA software. The black boxes indicate wild amino acids that would be affected by CRYBA4 high-risk pathogenic nsSNPs. The letter “h” denotes alpha helix; the letter “e” denotes extended strand, the letter “t” denotes beta turn and the letter “c” denotes random coil.

Evolutionary conservation is a key property of amino acid. ConSurf and SOPMA online tools were used to analyze the evolutionary conservation of CRYBA4 amino acid and protein structure. CRYBA4 protein structure is shown in Fig 4A, including the "Crystall" domain and "XTALbg" domain. According to the analysis of the Consurf online tool, there was a total of 60 evolutionary conserved positions with scores between 7 and 9 (Fig 4B). SOPMA online software predicted the secondary structure of the CRYBA4 protein. As shown in Fig 4C, CRYBA4 protein is composed of 196 amino acids, consisting of four secondary structures, 23 amino acids in the alpha helix (accounting for 11.73%), 45 amino acids in the extended strand (accounting for 22.96%), 22 amino acids in the beta turn (accounting for 11.22%), and 106 amino acids in the random coil (accounting for 54.08%).

Further analysis was carried out based on the pathogenicity prediction results. As shown in Fig 4 and Table 2, 39 wild-type amino acids can be replaced by 44 *CRYBA4* high-risk pathogenic nsSNPs, including 18 wild-type amino acids in the "Crystall" domain, 18 in the "XTALbg" domain and three in the other regions. Evolutionary conservation analysis showed that 29 amino acids were in conserved positions (12 amino acids got 9 points, seven amino acids got 8 points, five amino acids got 7 points, and five amino acids got 6 points). This suggested that they were highly conserved in evolution. At the same time, the protein secondary structure prediction indicated that one amino acid was located in the alpha helix, accounting for 2.56%; 11 amino acids in the extension strand, accounting for 28.21%; seven amino acids in beta turn, accounting for 17.95%; and 20 amino acids in random coil, accounting for 51.28%.

**Table 2:** Evolutionary conservativeness analyses and protein structure prediction of CRYBA4 high-risk pathogenic nsSNPs.

Amino acid change	Domain	Consurf score	SOPMA predicting secondary structure	Change of size	Change of charge	Change of Hydrophobicity	Other Influence
G10R	/	1	Random coil	W<M	Neutral->Positive	Decrease	The residue is located on the surface of the protein, Mutation of this residue can disturb interactions with other molecules or other parts of the protein. The torsion angles for this residue are unusual. Mutation into another residue will force the local backbone into an incorrect conformation and will disturb the local structure.
W12R	/	1	Random coil	W>M	Neutral->Positive	Decrease	
F22L	Crystall	8	Random coil	W>M			The mutation will cause a possible loss of external interactions.
R25W	Crystall	6	Random coil	W<M	Positive->Neutral	Increase	The residue is located on the surface of the protein, mutation of this residue can disturb interactions with other molecules or other parts of the protein.
R26W	Crystall	3	Random coil	W<M	Positive->Neutral	Increase	The residue is located on the surface of the protein, mutation of this residue can disturb interactions with other molecules or other parts of the protein.
R26P	Crystall	3	Random coil	W>M	Positive->Neutral	Increase	
E28K	Crystall	7	Extended strand	W<M	Negative->Positive		The residue is located on the surface of the protein, Mutation of this residue can disturb interactions with other molecules or other parts of the protein.
E32K	Crystall	6	Random coil	W<M	Negative->Positive		The residue is located on the surface of the protein, Mutation of this residue can disturb interactions with other molecules or other parts of the protein.
C33Y	Crystall	8	Random coil	W<M		Decrease	The wild-type residue was buried in the core of the protein. The mutant residue is bigger and probably will not fit.
R45L	Crystall	7	Random coil	W>M	Positive->Neutral	Increase	The mutation will cause a possible loss of external interactions.
S46Y	Crystall	9	Extended strand	W<M			The wild-type residue was buried in the core of the protein. The mutant residue is bigger and probably will not fit.
G56D	Crystall	5	Extended strand	W<M	Neutral->Negative	Decrease	The mutant residue introduces a charge in a buried residue which can lead to protein folding problems. The wild-type residue was buried in the core of the protein. The mutant residue is bigger and probably will not fit.
E58K	Crystall	7	Extended strand	W<M	Negative->Positive		The residue is located on the surface of the protein, Mutation of this residue can disturb interactions with other molecules or other parts of the protein.
**G64W**	**Crystall**	**9**	**Beta turn**	**W<M**		**Increase**	**The residue is located on the surface of the protein, mutation of this residue can disturb interactions with other molecules or other parts of the protein. The torsion angles for this residue are unusual. Mutation into another residue will force the local backbone into an incorrect conformation and will disturb the local structure.**
**L69P**	**Crystall**	**8**	**Extended strand**	**W>M**			**The mutation will cause an empty space in the core of the protein.**
R71L	Crystall	6	Beta turn	W>M	Positive->Neutral	Increase	The mutation will cause a possible loss of external interactions.
R71P	Crystall	6	Beta turn	W>M	Positive->Neutral	Increase	The mutation will cause a possible loss of external interactions.
E73K	Crystall	6	Random coil	W<M	Negative->Positive		The residue is located on the surface of the protein, mutation of this residue can disturb interactions with other molecules or other parts of the protein.
Y86S	Crystall	5	Random coil	W>M			
**S93P**	**Crystall**	**9**	**Extended strand**	**W<M**		**Increase**	**The wild-type residue was buried in the core of the protein. The mutant residue is bigger and probably will not fit. The mutation will cause loss of hydrogen bonds in the core of the protein and as a result disturb correct folding.**
**F94S**	**Crystall**	**5**	**Random coil**	**W>M**		**Decrease**	**The mutation will cause an empty space in the core of the protein. The mutation will cause loss of hydrophobic interactions in the core of the protein.**
R95W	Crystall	8	Random coil	W<M	Positive->Neutral	Increase	The residue is located on the surface of the protein, Mutation of this residue can disturb interactions with other molecules or other parts of the protein.
R95L	Crystall	8	Random coil	W>M	Positive->Neutral	Increase	The mutation will cause a possible loss of external interactions.
D104G	/	3	Random coil	W>M	Negative->Neutral	Increase	The mutation will cause a possible loss of external interactions.
E111K	XTALbg	9	Random coil	W<M	Negative->Positive		The wild-type residue was buried in the core of the protein. The mutant residue is bigger and probably will not fit.
E138G	XTALbg	5	Random coil	W>M	Negative->Neutral	Increase	The mutation will cause a possible loss of external interactions.
S141F	XTALbg	9	Extended strand	W<M		Increase	The wild-type residue was buried in the core of the protein. The mutant residue is bigger and probably will not fit. The mutation will cause loss of hydrogen bonds in the core of the protein and as a result disturb correct folding.
G147V	XTALbg	9	Beta turn	W<M		Increase	The wild-type residue was buried in the core of the protein. The mutant residue is bigger and probably will not fit. The torsion angles for this residue are unusual. Mutation into another residue will force the local backbone into an incorrect conformation and will disturb the local structure.
A148D	XTALbg	5	Random coil	W<M	Neutral->Negative	Decrease	The mutant residue introduces a charge in a buried residue which can lead to protein folding problems. The wild-type residue was buried in the core of the protein. The mutant residue is bigger and probably will not fit. The mutation will cause loss of hydrophobic interactions in the core of the protein.
V150D	XTALbg	9	Extended strand	W<M	Neutral->Negative	Decrease	The mutant residue introduces a charge in a buried residue which can lead to protein folding problems. The wild-type residue was buried in the core of the protein. The mutant residue is bigger and probably will not fit. The mutation will cause loss of hydrophobic interactions in the core of the protein.
C151F	XTALbg	5	Extended strand	W<M			The wild-type residue was buried in the core of the protein. The mutant residue is bigger and probably will not fit.
P155L	XTALbg	8	Beta turn	W<M			The residue is located on the surface of the protein, Mutation of this residue can disturb interactions with other molecules or other parts of the protein.
G156D	XTALbg	8	Beta turn	W<M	Neutral->Negative	Decrease	The residue is located on the surface of the protein, Mutation of this residue can disturb interactions with other molecules or other parts of the protein. The torsion angles for this residue are unusual. Mutation into another residue will force the local backbone into an incorrect conformation and will disturb the local structure.
G159R	XTALbg	9	Beta turn	W<M	Neutral->Positive	Decrease	The residue is located on the surface of the protein, Mutation of this residue can disturb interactions with other molecules or other parts of the protein. The torsion angles for this residue are unusual. Mutation into another residue will force the local backbone into an incorrect conformation and will disturb the local structure.
Q161H	XTALbg	9	Extended strand	W<M			The wild-type residue was buried in the core of the protein. The mutant residue is bigger and probably will not fit.
Y162C	XTALbg	7	Extended strand	W>M		Increase	The mutation will cause an empty space in the core of the protein. The mutation will cause loss of hydrogen bonds in the core of the protein and as a result disturb correct folding.
G171S	XTALbg	7	Beta turn	W<M			The wild-type residue was buried in the core of the protein. The mutant residue is bigger and probably will not fit. The torsion angles for this residue are unusual. Mutation into another residue will force the local backbone into an incorrect conformation and will disturb the local structure.
Y173C	XTALbg	4	Random coil	W>M		Increase	The mutation will cause an empty space in the core of the protein. The mutation will cause loss of hydrogen bonds in the core of the protein and as a result disturb correct folding.
R177W	XTALbg	4	Random coil	W<M	Positive->Neutral	Increase	The mutant residue is bigger, this might lead to bumps. The mutation introduces a more hydrophobic residue at this position. This can result in loss of hydrogen bonds and/or disturb correct folding.
E178K	XTALbg	5	Random coil	W<M	Negative->Positive		The wild-type residue was buried in the core of the protein. The mutant residue is bigger and probably will not fit.
W179R	XTALbg	3	Random coil	W>M	Neutral->Positive	Decrease	Hydrophobic interactions, either in the core of the protein or on the surface, will be lost.
R192H	XTALbg	9	Alpha helix	W>M	Positive->Neutral		The mutation will cause a possible loss of external interactions.
R192P	XTALbg	9	Alpha helix	W>M	Positive->Neutral	Increase	The mutation will cause a possible loss of external interactions.
R192C	XTALbg	9	Alpha helix	W>M	Positive->Neutral	Increase	The mutation will cause a possible loss of external interactions.

The bold black font represents the nsSNP reported in the literature. W: wild type M: mutant type

### Protein property analysis

**Fig 5:**
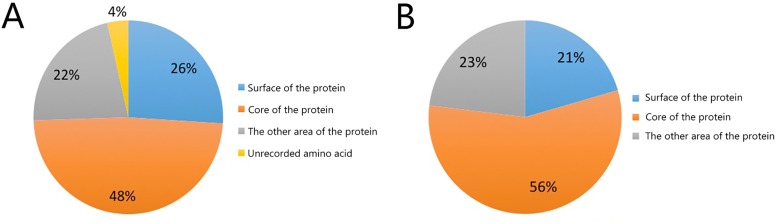
The solvent accessible surface areas of the protein result calculated by GETAREA software. A. The solvent accessible surface areas of all 189 amino acids. B. The solvent accessible surface areas of 39 CRYBA4 high-risk pathogenic nsSNPs related amino acids.

GETAREA and HOPE online tools were used to analyze the solvent accessible surface areas, physical and chemical properties, and changes in protein structure. Using "3lwk.1 crystal structure of human Beta-crystallin A4" as a template, 39 "*CRYBA4* high-risk pathogenic nsSNP" related amino acids were analyzed by GETAREA and HOPE online software. GETAREA calculated the solvent-accessible surface areas of the protein (Fig 5). Except for seven (4%) unrecorded amino acids, the software calculated the solvent-accessible surface areas of 189 amino acids. There were 51 (26%) amino acids in the surface of the protein, 95 (48%) in the core of the protein and 43 (22%) in other parts of the protein. Among the 39 *CRYBA4* high-risk pathogenic nsSNP related amino acids, there were eight (21%) amino acids in the surface of the protein, 22 (56%) in the core of the protein and nine (23%) in other parts of the protein. This suggested that mutations in the core of the protein were more likely to have harmful effects on protein structure and function.

HOPE predicted the changes of amino acids before and after mutation on the physical and chemical properties, hydrophobicity, spatial structure, and function of protein (Table 2). All 44 *CRYBA4* high-risk pathogenic nsSNPs caused changes in size of the amino acids (18 smaller and 26 larger). There were 28 nsSNPs that caused change of charge. Also, 29 nsSNPs changes the hydrophobicity of the amino acids (10 lower and 19 higher). The changes of physical and chemical properties of these sites before and after amino acid mutation led to changes in protein structure and interaction between structural domains and other molecules, affecting the protein function.

## Discussion

In a genome, nsSNPs account for about 50% of allele variation of all hereditary human diseases [37]. Identifying nsSNPs responsible for specific phenotypes using molecular approaches is expensive and time-consuming [38], and so bioinformatics predicting approaches can help in narrowing down the number of high-risk pathogenic nsSNPs to be screened in genetic association studies, and in a better understanding of the function and structure of protein products.

However, the performances of different bioinformatics tools under various conditions were different. It is important to choose tools with good performance. In order to objectively evaluate performances of different tools, Li. et al [18] compared 12 performance measures of 23 methods based on three independent benchmark datasets. Luxembourg. et al [19] used *SERPINC1* missense mutations to test 12 tools. According to their tentative guidance for optimal tool section, we chose six bioinformatics tools as pathogenic predictors (Mutpred2, PANTHER-PSEP, PhD-SNP, PolyPhen 2.0, PROVEAN and SIFT) for their high accuracy and frequency of use.

In this study, 44 nsSNPs that were predicted to be pathogenic by all software tools were defined as “*CRYBA4* high-risk pathogenic nsSNPs”. In order to further narrow down the number of possible pathogenic nsSNPs, the prediction of protein stability, the evolutionary conservation of amino acids, the physical and chemical properties, and changes of protein structure after mutations were analyzed by I-Mutant 2.0, Mupro, INPS, ConSurf, SOPMA, GETAREA and HOPE online software tools.

Protein stability is an important characteristic influencing the function, activity and regulation of proteins. Changes in protein stability are usually accompanied by changes in free energy (ΔΔG). When ΔΔG < 0, the stability decreases [30–32]. It has been reported that the breakdown of lens microarchitecture and the aggregation of crystallins can cause cataracts [39,40]. Human γD crystallin (HγDC) is one of the most abundant crystallins in the central lens nucleus, and its thermodynamic stability and aggregation are associated with the development of age-related cataracts [10]. Aguayo-Ortiz R et al, [41] have used alchemical free energy calculations to predict changes in the thermodynamic stability (ΔΔG) of 10 alanine-scanning variants and 12 HγDC mutations associated with the development of congenital cataracts. These results showed that changes in ΔΔG are associated with the significant position of the motif, thus affecting thermodynamic stability. Our analyses clearly show that 68.2% (30/44) of the *CRYBA4* high-risk pathogenic nsSNPs could lead to a decrease in protein stability, and only two of these have been reported in published literature. These mutations of single or multiple amino acids can cause changes and damage in hydrophobicity, protein folding, main chain tension, and electrostatic force, leading to changes in protein stability [42]. We recommend further investigations on nsSNPs that lead to a decrease in protein stability. In addition, we recommend conducting experiments about molecular dynamics in order to investigate the effect of mutations on the stability of protein structure.

In addition to analyzing changes in free energy after mutations, the effect of mutation on protein stability is still based on changes of protein structure. Highly evolutionary conserved amino acids are important parts of secondary structure and domains. Mutations that cause substitution of highly conserved amino acids may change the secondary structure and interaction between structural domains and other molecules, which would affect protein function. Our study shows that 74.4% (29/39) of the amino acids related to “*CRYBA4* high-risk pathogenic nsSNPs” are conserved and 92.3% (36/39) of them are in important functional domains. The high-risk pathogenic nsSNPs leads to changes in size, charge and hydrophobicity of most amino acids. According to Table 2, all 44 *CRYBA4* high-risk pathogenic nsSNPs led to changes in amino acid size. 63.6% (28/44) of the *CRYBA4* high-risk pathogenic nsSNPs changed the charge through substitution of amino acids and 65.9% (29/44) of nsSNPs changed the hydrophobicity. Thirteen residues are located on the surface of the *CRYBA4* protein. Mutation in these locations may affect the disturbance interactions with other molecules or other parts of the protein. We used String (https://string-db.org) software and found three molecules (Beta-crystallin B1, CRYBB1; Beta-crystallin B2, CRYBB2 and DNA damage-binding protein 2, DDB2) that had interactions with CRYBA4. CRYBA4, CRYBB1 and CRYBB2 all belong to crystallin family. These three proteins have similar sequences and are co-expressed in humans and other species. DDB2 is required for DNA repair. It can bind to DNA damage-binding protein 1 (DDB1) to form the UV-damaged DNA-binding protein complex (the UV-DDB complex). It has been reported that tritiated water exposure disrupts myofibril structure and induces misregulation of eye opacity through the mutation of *CRYBA4* and DNA repair genes in early zebrafish life stages [43]. However, more experiments are needed to fully confirm the connection between CRYBA4 protein and DDB2 protein. Our study suggests that nsSNPs in conserved positions may affect the function and properties of protein by changing the protein structure.

Our study provides predictive results of pathogenicity, as well as analysis results of protein properties and structural changes by nsSNPs. In order to test the accuracy of our predictive results, we conducted the comparison between theoretical prediction results and experimental studies in literature. As we found, the accuracy of prediction results was good (Table 3). For example, we predicted that G64W cause unusual torsion angles, thus forcing the local backbone into an incorrect conformation and disturbing the local structure and interactions with other molecules. According to Li et al[44], CRYBA4 p.G64W is prone to form inclusion body when expressed in E. coli which indicated that the p.G64W mutant might affect its folding properties of CRYBA4. Besides, G64W increases self-interaction in vitro and in mammalian cell. CRYBA4 p.G64W is less stable because it can affect CRYBA4 folding and block its interaction with CRYBB1.However, we also found that the literature data was insufficient. Further work is required to verify the accuracy of bioinformatics analysis.

**Table 3:** Comparison between theoretical prediction results and biophysical experimental data available in the literature on beta crystallins.

	A9V	G64W	Y67N	L69P	S93P	F94S
MutPred2	0.227	0.937	0.87	0.956	0.921	0.803
PANTHER	probably benign	probably damaging	probably benign	probably damaging	probably damaging	probably damaging
PROVEAN	Neutral	Deleterious	Deleterious	Deleterious	Deleterious	Deleterious
SIFT	Tolerated	Damaging	Damaging	Damaging	Damaging	Damaging
PolyPhen-2	benign	probably damaging	possibly damaging	probably damaging	probably damaging	probably damaging
PhD-SNP	Neutral	Disease	Disease	Disease	Disease	Disease
I-Mutation2.0	-1.06	0.17	-0.24	-0.36	0.36	-1.38
Mupro	-0.28	-0.20	-1.12	-2.18	-0.98	-1.86
INPS	0.84	-0.81	-1.84	-3.16	-0.92	-2.60
Consurf score	1	9	6	8	9	5
Change of size	W<M	W<M	W>M	W>M	W<M	W>M
Change of charge	None	None	None	None	None	None
Change of Hydrophobicity	Increase	Decrease	None	Increase	Decrease	
Influence	1. Disturb interactions with other molecules or other parts of the protein.	1. Disturb interactions with other molecules. 2. Unusual torsion angles. 3. Force the local backbone into an incorrect conformation and will disturb the local structure	1. Cause empty space in the core of the protein. 2. Cause loss of hydrophobic interactions in the core of the protein	1. Cause an empty space in the core of the protein	1. The mutant residue is bigger and probably will not fit 2. Cause loss of hydrogen bonds in the core of the protein and as a result disturb correct folding.	1. Cause an empty space in the core of the protein. 2. Cause loss of hydrophobic interactions in the core of the protein
Experimental findings in published literature	1. This mutation is cosegregated with congenital cataracts within the family. 2. There is no further experimental findings.	1. CRYBA4 p.G64W is prone to form inclusion body when expressed in E. coli which indicated that the p.G64W mutant might affect its folding properties of CRYBA4. 2. G64W affects its folding in E. coli. 3. G64W increases self-interaction in vitro and in mammalian cell. 4. CRYBA4 p.G64W is less stable than WT. 5. G64W blocks its interaction with CRYBB1. 6. G64W maintains its interaction with CRYAA.	1. CRYBA4 p.Y67N was found in two cases affected with bilateral nuclear cataract but was not found in normal controls. 2. The mutation CRYBA4 p.Y67N located in the neighboring β strand of the N-terminal domain. 3. As this protein is functional in its multimeric form, the increased flexibility in the mutant affects the stability of the oligomer as well as interactions with other partner proteins.	1. This mutation is cosegregated with congenital cataracts within the family. 2. It is predicted here to disrupt the β-sheet structure in CRYBA4. 3. Protein folding would consequently be impaired, most probably leading to a structure with reduced stability in the mutant.	1. This mutation is cosegregated with congenital cataracts within the family. 2. There is no further experimental findings.	1. This mutation is cosegregated with congenital cataracts within the family. 2. There is no further experimental findings.
Reference	Zhai, 2017[45]	Li, 2019[44]	Kumar,2013[15]	Billingsley,2006[11] Kumar,2013[15]	Li, 2018[17]	Billingsley,2006[11]

By using our predictive methods, we obtained four important nsSNPs (c.283C>T, p.R95W; c.449T>A, p.V150D; c.475G>A, p.G159R; c.575G>C, p.R192P) from 44 *CRYBA4* high-risk pathogenic nsSNPs to focus on because: (i) the four nsSNPs were predicted to be pathogenic by all six predicting tools; (ii) according to three protein stability analyzing tools, they could all lead to ΔΔG < 0, resulting in protein stability decline; (iii) the results of evolutionary conservation analyzing tools showed that the four nsSNPs were all in highly conserved sites; and (iv) protein structural analyzing tools indicated that all four nsSNPs could cause changes in the size, charge and hydrophobicity of CRYBA4 protein.

Admittedly, there were several limitations of this study. The first limitation of this study is that it describes a comparative analysis using public data. We have found and reported ADCC pedigree with SNPs of the *CRYBA4* gene. In our previous study, we found a new *CRYBA4* nsSNP (c.169T>C, p.F57L) in an ADCC pedigree. We used methods in our article to predict the pathogenicity of this nsSNP and predicted it to be pathogenic. We have conducted experiments to confirm its pathogenicity. We used the methods mentioned to predict its pathogenicity and conducted experiments to explore the relationship between the SNPs and ADCC. Second, although we tried to improve the accuracy of our prediction by using multiple pathogenic predictors and analyzing their possible impacts on protein structure and properties, there were still several pitfalls for their false positive and negative predictions. The six bioinformatics tools were chosen based on their high accuracy and frequency of use. However, the six tools in the first stage use somewhat overlapping concepts to make their predictions. They rely heavily on conservation and other easy methods to calculate differences between amino acids. More information on ADCC pedigree should be collected to train and improve the accuracy of predicting tools. Third, the population allele frequencies should be taken into consideration. According to our clinical experience, mutations of the same gene can result in distinct cataract phenotypes. They may be neglected if mild opacity does not seriously affect visual acuity and appearance. Therefore, the disease may be missed and the incidence of congenital cataracts would not be reported. This problem requires the attention of ophthalmologists and researchers, and is one of the main purposes of our research.

## Conclusions

In conclusion, our study provides not only predictive results of pathogenicity, but also the analysis of protein properties and structural changes by nsSNPs, allowing us to select 44 *CRYBA4* high-risk pathogenic nsSNPs that are likely to have functional impact on the *CRYBA4* gene and contribute to the pathogenesis of ADCC. Among these, there are four nsSNPs (c.283C>T, p.R95W; c.449T>A, p.V150D; c.475G>A, p.G159R; c.575G>C, p.R192P) that should be focused on because of their high potential pathogenicity predicted using bioinformatics tools. This computational study provides a theoretical basis for the molecular mechanism of ADCC caused by *CRYBA4* gene mutation. This cost-effective and easy method of analyzing the predicted deleterious nsSNPs of the *CRYBA4* gene has important reference value and will contribute to further studies of the treatment of *CRYBA4* related ADCC.

## Supporting information

S1 File RAW Data of *CRYBA4* nsSNPs predictions. (XLSX)
